# P-31. Outcomes Associated with the 2024 Blood Culture Shortage at a Large Academic Medical Center

**DOI:** 10.1093/ofid/ofaf695.260

**Published:** 2026-01-11

**Authors:** Joseph B Ladines-Lim, Leigh Cressman, Kyle Rodino, Laurel Glaser, Kathleen Degnan, Michael Z David

**Affiliations:** Penn Medicine, Philadelphia, PA; Penn Medicine, Philadelphia, PA; University of Pennsylvania, Philadelphia, PA; University of Pennsylvania, Philadelphia, PA; University of Pennsylvania Perelman School of Medicine, Philadelphia, Pennsylvania; University of Pennsylvania Perelman School of Medicine, Philadelphia, Pennsylvania

## Abstract

**Background:**

The Infectious Diseases Society of America and American Society for Microbiology recommend 2–3 blood culture sets in adults with suspected bloodstream infection. In June 2024, a national shortage of BACTEC blood culture bottles led our institution, a large, urban academic medical center, to restrict blood cultures to one set per patient every 24 hours from June 26 to December 23, 2024. While other institutions have described stewardship efforts during the shortage, little is known about its impact on clinical outcomes. We conducted a retrospective, quasi-experimental analysis to evaluate these effects.

**Figure:**
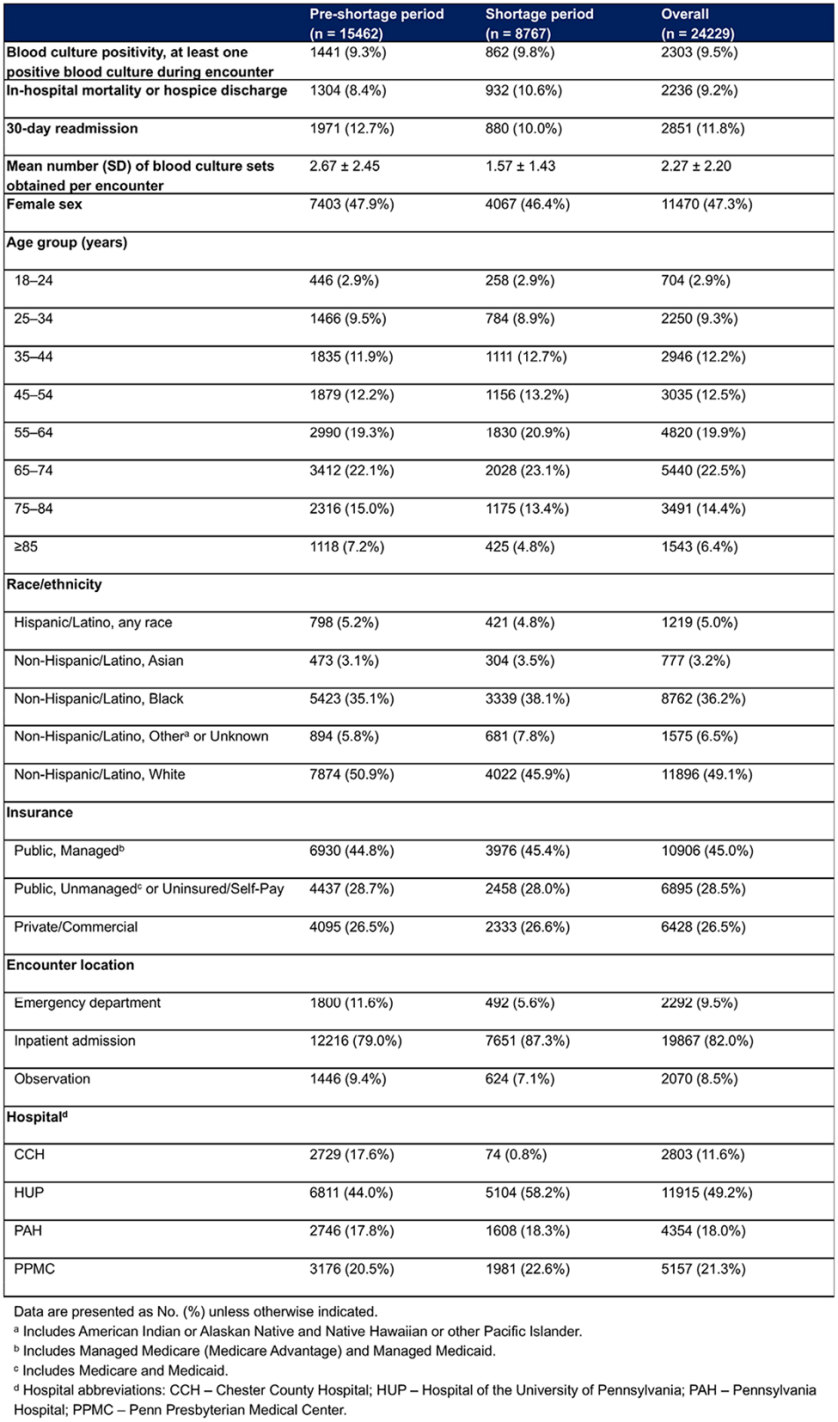
Characteristics of Encounters with ≥1 Blood Culture Set Obtained During the Pre-Shortage (December 24, 2023–June 25, 2024) and Shortage (June 25, 2024–December 23, 2024) Period

**Figure:**
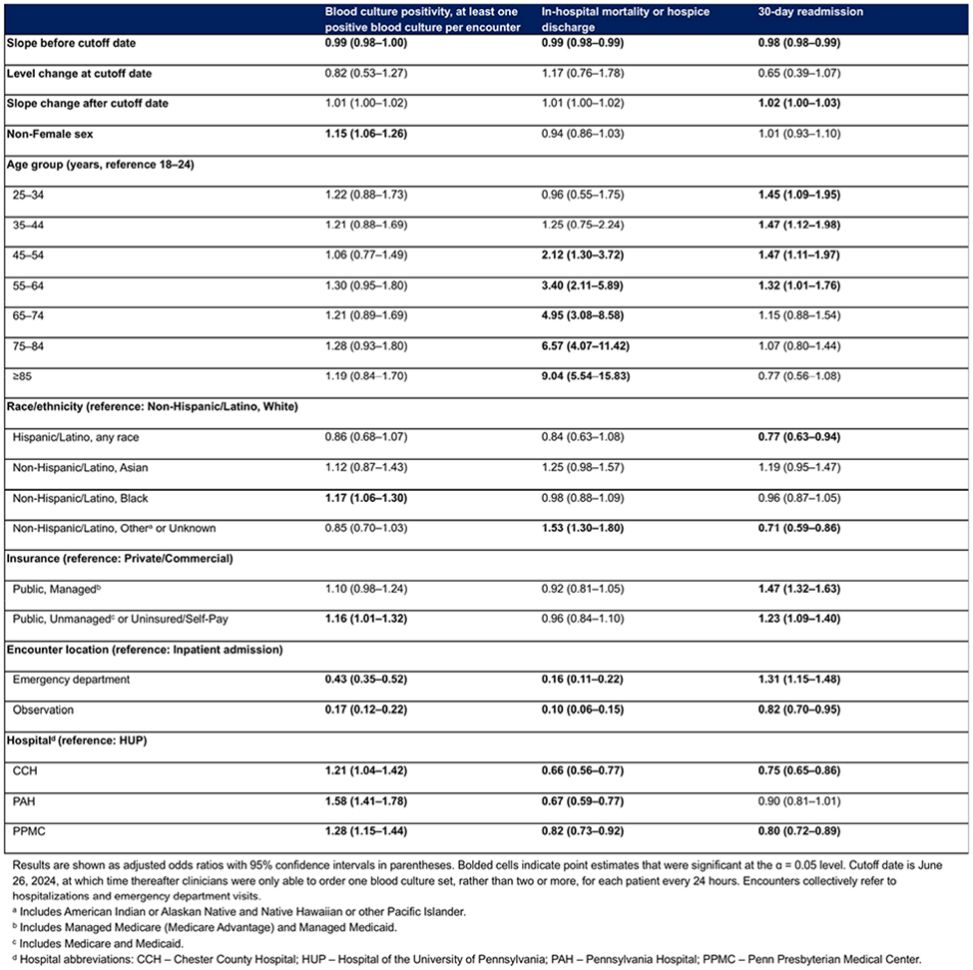
Association Between Changes in Main Outcomes, Blood Culture Shortage Period, and Covariates

**Methods:**

We analyzed hospitalizations and emergency department (ED) encounters with ≥1 blood culture from December 24, 2023 to December 23, 2024. Outcomes included blood culture positivity, in-hospital mortality or hospice discharge, and 30-day readmission. We used segmented regression models in an interrupted time series analysis to assess changes at the June 26, 2024 cutoff, adjusting for covariates.

**Figure:**
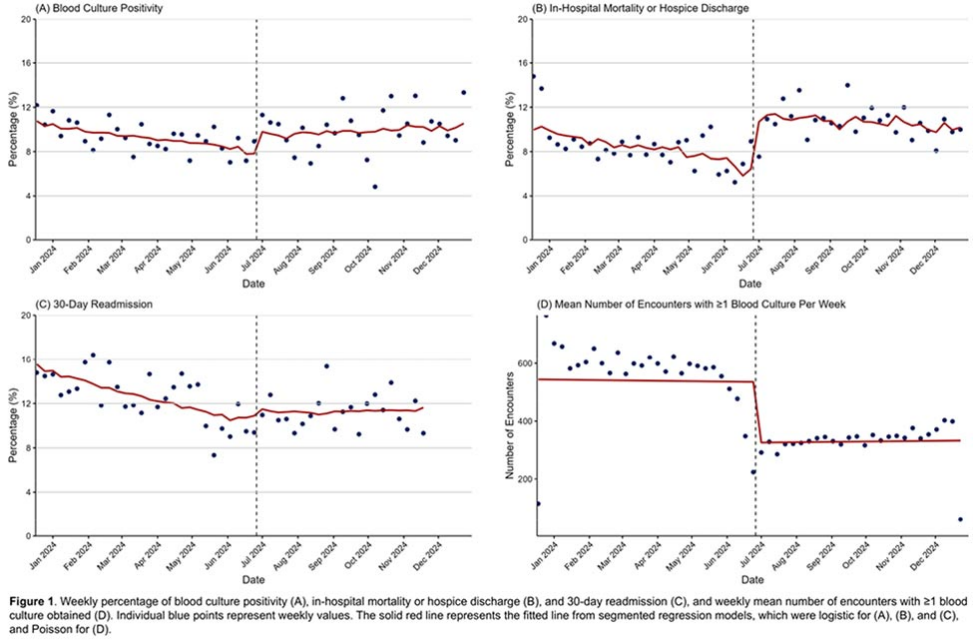

**Results:**

We identified 24229 encounters with ≥1 blood culture. Patient characteristics were similar pre- and during-restriction, with decreased encounters with ≥1 blood culture in the ED and one hospital site during the restriction period (Table 1). All outcomes declined slowly prior to the shortage; only 30-day readmission had a minor slope increase during restriction (Table 2 and Figure 1A–C). Encounters with ≥1 culture decreased markedly (Figure 1D). Regression identified various sociodemographic predictors of outcomes (Table 2). Of note, inpatient admission and our institution’s flagship hospital, to which the other hospitals often refer patients for higher level of care, were associated with all outcomes (Table 2).

**Conclusion:**

Blood culture restriction had marginal impact on outcomes but markedly reduced the number of cultures obtained and seemed to shift testing to inpatients and our flagship hospital. These shifts may have influenced outcomes as suggested by regression modeling, though this remains speculative without further study. Limitations include retrospective, single-center design and lack of other potential covariate data (e.g. patient comorbidities). Future work will also examine other key outcomes, including intensive care admission and length of stay.

**Disclosures:**

All Authors: No reported disclosures

